# Transplantation of Cryopreserved Olfactory Ensheathing Cells Restores Loss of Functions in an Experimental Model

**DOI:** 10.1177/09636897231199319

**Published:** 2023-09-29

**Authors:** Kamile Minkelyte, Daqing Li, Ying Li, Ahmed Ibrahim

**Affiliations:** 1 Spinal Repair Unit, Department of Brain Repair and Rehabilitation, UCL Institute of Neurology, Queen Square, London, UK; 2 Imperial College Healthcare NHS Trust, London, UK

**Keywords:** olfactory ensheathing cells, transplantation, allograft, spinal cord injury, spinal roots; cryopreservation

## Abstract

In the past decades, the properties of olfactory ensheathing cells (OECs) have been widely investigated. Studies have shown that transplantation of OECs cultured from the olfactory bulb mediates axonal regeneration, remyelination and restores lost functions in experimental central nervous system (CNS) injury models. Autologously sourcing the cells from the nasal mucosa or the olfactory bulb to treat patients with spinal cord injuries would be ideal, but the cell yield achieved may be inadequate to cover the surface area of the lesions typically encountered in human spinal cord contusion injuries. Therefore, banking allogenic cryopreserved olfactory bulb cells from donors or generating cell lines could provide a marked increase in cell stock available for transplantation. This study is undertaken in two control and two intervention groups. The control groups have lesions alone and lesions with collagen gel but without cells. The intervention groups have either transplantation of primary cultured olfactory bulb OECs (bOECs) encapsulated in collagen gel or cryopreserved bulb OECs (CbOECs) encapsulated in collagen gel. Here, we report that transplantation of cryopreserved rat bOECs encapsulated in collagen restored the loss of function in a vertical climbing test in a unilateral C6-T1 dorsal root injury model. The loss of function returns in 80% of rats with injuries in about 3 weeks comparable to that we observed after transplantation of primary cultured bOECs. The regeneration axons induced by the transplant are identified by neurofilament antibodies and ensheathed by OECs. Our results indicate that cryopreserved OECs retain their properties of inducing axon regeneration and restoring loss of function in the experimental model. This is a step forward to translate the research into future clinical applications.

## Introduction

Cell transplantation is one of the most promising strategies for the repair of central nervous system (CNS) injuries. Many studies have shown that transplantation of olfactory ensheathing cells (OECs) can induce axon regeneration, remyelination, and restoration of function in experimental spinal cord injuries^1–12^. Our previous studies showed that transplantation of cultured bulbar OECs in rats to bridge transected dorsal roots to the spinal cords induced the regeneration of axons and restored directed forepaw reaching and vertical climbing functions^6,13^.

From a clinical point of view, obtaining a biopsy or harvesting of a patient’s olfactory bulb necessitates a transnasal endoscopic or transcranial approach, which is associated with significant morbidity such as bleeding, meningitis, stroke, paralysis, and unilateral anosmia^14^. In addition, the yield of OECs acquired from the biopsy could be unpredictable and the cell culture failure would be costly. In this study, we set out to investigate whether a bank of cryopreserved allogeneic cells or cell lines can restore neurological function in an experimental injury model.

Transplanted allogeneic cells would be vulnerable to an immune reaction and would have the potential to be rejected by the recipient. Several studies using immune-incompatible grafts with an immunosuppressant drug have shown that xenografted OECs induce axon regeneration and remyelination in the spinal cord^15–19^.

Allografts have been studied experimentally, for example, treatment of corneal blindness using bone marrow mesenchymal stem cells, long-term tolerance of islet allografts induced by apoptotic donor leukocytes, and treatment of bone fractures^20–23^. Allograft transplants have also been used clinically, for example, for cartilage repair, renal injury, bone allografts, liver transplant, and nerve injury^24–28^. Although allografts have been used clinically^29,30^, allografting banked cells are not yet applied clinically to treat patients’ CNS injuries.

Some research has been done on the effects of cryopreservation on OECs. One study showed that bOECs are resilient to cryopreservation^31^. And others have shown that after cryopreservation cells maintain their functions and continue to have a positive impact on spinal cord injury recovery^32,33^.

Overall, there are limited studies evaluating the effects of cryopreservation on OECs and it may depend on a variety of factors, including the cryopreservation protocol used, the length of time the cells are stored, and the specific characteristics of the OECs being preserved. Here, we report that using cryopreserved cell cultures from rat olfactory bulbs can induce regeneration and restore loss of function in an experimental model.

## Materials and Methods

All animals were used under the UK Home Office regulations for the care and use of laboratory animals, the UK Animals (Scientific Procedures) Act 1986, with the ethical approval of the University College London Institute of Neurology. Adult female Sprague–Dawley (SD) rats at a body weight of 150–200 g were purchased from Charles River, UK. Adult green rats supplied by UCL School of Pharmacy as gifts were used to obtain the cells. A total of 29 animals were used in this study.

### Experimental Design

#### Control groups

Two groups of controls were included in the study: Group 1, dorsal root transection alone: rats received C6-T1 unilateral dorsal roots transection (*n* = 6) and Group 2, rats with dorsal root transection and transplanted collagen gel without cells (*n* = 5).

#### Transplant groups

Two treatment groups were included in this study. Group 1, rats with the dorsal root transection and transplantation of primary cultured bOECs encapsulated in collagen gel (*n* = 8). This group was used as a positive control as it has been well-documented that the bOECs led to functional improvement. Group 2, rats with the dorsal root transection and transplantation of CbOECs encapsulated in collagen gel (*n* = 10).

### Cell Preparation

#### BOECs culture

The cells were cultured from the olfactory bulbs of SD rats (a β-actin–GFP reporter line—kindly offered to us by Professor James Phillips from UCL School of Pharmacy). The detailed protocol was given in our previous study^3^. Briefly, under aseptic conditions, the nerve, and glomerular layers of the olfactory bulbs from adult SD rats were dissected out, dissociated using 0.25% trypsin (Thermo Fisher, USA), and tissue from about one bulb per dish was seeded onto poly-d-lysine (PDL; Sigma, Japan) coated 35-mm Nunc dishes. The cells were then cultured in complete media consisting of DMEM/F-12 media supplemented with 10% fetal bovine serum and +1% PenStrep (10,000 U/ml penicillin, 10,000 µg/ml streptomycin; Gibco, USA). All cultures were maintained in a humidified incubator enriched with 5% CO2 at 37°C; the medium was replaced every other day for 14 days when the culture became confluent.

#### Cell cryopreservation and recovery procedure

After 14 days in culture, the cells were washed with Hanks’ Balanced Salt Solution (HBSS) and then 0.25% trypsin/Ethylenediaminetetraacetic acid (EDTA (TE)) was added for 15 min at 37°C. The complete media was added to stop the reaction, the cells were pooled into a falcon tube and then centrifuged at 350 × *g* for 5 min. The supernatant was discarded, and the cell pellet was resuspended in a cell culture freezing media (Thermo Fisher). Aliquots of the cell suspension at a density of approximately 1 × 10^6^ were dispensed into cryogenic storage vials which were transferred into a controlled rate freezing apparatus (Mr. Frosty, Thermo Fisher, USA) and stored at −80°C overnight. The cryogenic storage vials were then transferred and stored in liquid nitrogen. The cryopreserved cells were thawed from liquid nitrogen after 4 weeks by removing them from the liquid nitrogen and rapidly thawing them to 37°C. The cell suspension was centrifuged at 350 × *g* for 5 min, and the supernatant was removed. Fresh media was added to resuspend the cells and they were then replated onto the 35-mm Nunc culture dishes at a density of 1 × 10^6^/ml. The cells were cultured for a further 2 days before being encapsulated in collagen gel.

#### Encapsulation of bOECs in collagen

The details of cell/collagen preparation have been described in our previous publication (Collins et al.^13^). Briefly, the cells were incubated in 0.25% TE to be lifted from the bottom of the culture dishes. The TE was inactivated by adding complete media, and the cells were collected and triturated into a single-cell suspension. The cells were encapsulated by mixing a cell suspension with type 1 rat tail collagen (Corning, USA), 1M NaOH, d.H_2_O, and Modified Eagle’s Medium (MEM, 10×; Sigma-Aldrich, Missouri, USA). A volume of 250 µl of cell/collagen mixture was deposited on a 35-mm culture dish and placed into an incubator where it polymerized to form a gel matrix. The final collagen concentration was about 4.8 mg/ml. Once the gel became firm, it was submerged in the complete media until use. The media was changed every other day as with the cell culture. Each collagen gel contained approximately 2 × 10^5^ cells with a diameter of around 1 cm and a thickness of around 1 mm. The cells were cultured in the collagen gel for a further 2–3 days and were trimmed into approximately 2-mm^2^ pieces before they were transplanted.

### Surgery

#### Dorsal root transection alone

Unilateral transection of four dorsal rootlets was carried out as described in our previous study^34^. Briefly, a skin incision was made along the dorsal midline under isoflurane anesthesia and analgesia (Vetergesic 0.1-0.05mg/kg; Ceva, France). The prominent T2 process was located and hemilaminectomies were performed from C4 to T2, and the dura was incised with a pair of micro scissors to reveal the dorsal roots. The rootlets of C6, C7, C8, and T1 were transected with micro scissors as close as possible to the spinal cord in a plane approximately perpendicular to their entry into the spinal cord. The cut rootlets were re-apposed and held in place with fibrin glue (Tisseel Kit; Baxter, Thetford, UK).

#### Dorsal root transection with collagen gel transplant

The surgical procedure was the same as the injury alone. The collagen gel without the cells was immediately applied between the cut ends of the rootlets and their original entry point on the spinal cord and held in place with the fibrin glue after C6-T1 unilateral dorsal roots were transected.

#### Dorsal root transection with primary cultured bOECs encapsulated in collagen gels

The same surgical procedure was carried out as in the control groups. The rats received bOECs encapsulated within a collagen gel immediately after four unilateral dorsal rootlets were transected. The transplant was applied between the cut ends of the rootlets and their original entry point on the spinal cord and held in place with fibrin glue (Tisseel Kit, Baxter, Thetford, UK).

#### Dorsal root transection with cryopreserved bOECs encapsulated in collagen gels

The same surgical procedure was carried out as in the dorsal transection with primary cultured bOECs encapsulated in collagen gels: The rats received CbOECs after four unilateral dorsal rootlets were transected. The transplant was applied between the cut ends of the rootlets and their original entry point on the spinal cord and held in place with the fibrin glue.

#### Post-operative care

After the animal received dorsal root transection or transplantation, the overlying muscles and skin were sutured in layers (Vicryl Plus, Ethicon; VWR, UK). The animals were placed in a warm cage to recover from anesthesia before returning to their home cage. A wet diet and postoperative pain relief (0.05 mg/kg buprenorphine, s.c. daily for 3 days) were given. Bitter paste (Henry Schein, USA) was applied to the forepaw on the operated side every other day for the first 2 weeks to prevent autotomy. Autotomy was observed in five animals, one in the control and four in the transplanted groups. If the autotomy occurred to the extent of involving two joints in one toe, or past the nail, in two toes, the animals were culled humanely (under the guidance of the Project License). The rat housing room was maintained between 20°C and 22°C under standard lighting conditions (12:12 light–dark cycle) with food and water available *ad libitum*.

### Function Test and Analysis

**Figure 1.**
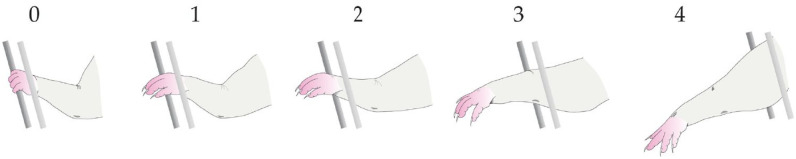
Error grading scheme. Error grading scheme for the degree of accuracy in the forepaw locating and grasping the grid bars. 0, successful grasp; 1, paw reaches grid level but no grasping; 2, protrudes through the grid as far as the wrist; 3, as far as the elbow; 4, as far as the axilla. Figure adapted from Minkelyte et al.^34^

The details of the test procedure have been described in our previous studies^6,34^. The assessment is based on two parameters—Grasping Error Percentage and Foot Fault Score. Briefly, the rats were placed on the lower bars of a 1-m vertical grid at 15° inclination and allowed to climb up the rungs to the top. This was repeated twice a week, 1 week before surgery and for up to 6 weeks post-surgery. If a rat grasped the bars successfully with all attempts for one complete climb, it scored 0% error; if it failed to grasp the bars with all attempts, it scored 100% error. Unsuccessful grasps were graded from 1 to 4 in increasing order of severity depending on how far the forelimb protruded through the grid (1, paw reaches grid level but no grasping; 2, protrudes through the grid as far as the wrist; 3, as far as the elbow; 4, as far as the axilla. Shown in Fig. 1). Video recordings were made of three successive climbs and analyzed by two people blindly.

### Immunohistochemistry

#### For *in vitro* study

Thawed cells were cultured for 2 days and then fixed for 30 min with 4% paraformaldehyde (PFA) in 0.1M phosphate buffer (4% PFA; Sigma-Aldrich), washed in 0.01M phosphate-buffered saline (PBS), and incubated in a blocking solution containing 2% skimmed milk (Oxoid LP6031, Thermo Fisher, USA) + 1% Triton X-100 (Fisher Bioreagents, BP151-500, Fairlawn, NJ, USA) for 60 min. The cells were incubated overnight at 4°C with a cocktail of primary antibodies: mouse monoclonal antibody against low-affinity nerve growth factor receptor (P75, 1:500; Millipore, Temecula, CA, USA) and rabbit polyclonal antihuman fibronectin (FN, 1:1,000; Dako, Glostrup, Denmark). After washing in PBS, the cells were incubated with fluorophore-conjugated secondary antibodies (goat anti-mouse Alexa 488 and goat anti-rabbit Alexa 546, both 1:500; Molecular Probes, Invitrogen) for an hour, washed in PBS, counterstained, and mounted using Prolong Gold antifade reagent containing the nuclear dye 40, 6-diamidino-2-phenylindole (DAPI, 0.4 μg/ml; Thermo Fisher Scientific, Massachusettes, USA). The culture area was divided into four quadrants on each culture dish, and four images sized 1.02 mm × 0.76 mm in each quadrant through each of the three fluorescent channels (red, blue, and green) were captured on a Nikon Eclipse 55i microscope (Japan Optics, Japan) 100× magnification. The total cell number and the subpopulation of OECs and olfactory nerve fibroblasts (ONFs) in the sampled area were counted with the Fiji software (based on ImageJ developed by NIH [National Institutes of Health]).

#### For *in vivo* study

Six weeks after surgery, the rats were terminally anesthetized by an overdose of CO_2_ and transcardially perfused with 0.01M PBS followed by 500 ml of 4% PFA for 30 min. The vertebral columns were dissected from the craniocervical junction to the upper thoracic level and left to harden in the same fixative for 2–3 days at 4°C. The spinal cord and associated roots were carefully dissected under a dissecting microscope to preserve the continuity across the dorsal roots and transplants to the spinal cord. The tissues were placed sequentially into 10% and 20% sucrose solution until they sunk. Serial frozen sections were cut on a cryostat (Leica CM3050, Leica Biosystems, Germany) after the tissue block was mounted with an embedding compound Cryo-M-Bed (Fisher Scientific, Massachusettes, USA) and frozen in the Cryostat chamber at −20°C until hardened. Horizontal plane sections of 16 µm at the levels of C6-T1 were cut and thaw-mounted onto slides. For immunohistochemistry, all sections were fixed with 4% PFA for 30 min and then washed with PBS. They were then incubated in a 2% milk PBS-blocking solution before applying antibodies diluted in a 2% blocking solution. The sections were washed in PBS for 3 × 10 min. For double immunostaining, sections were incubated in the primary antibodies (see Table 1). Fluorescent images were visualized and captured using a TCS SP1 Leica confocal microscope (Leica Biosystems, Germany). The laser intensity, pinhole sizes, and sensitivity of the photomultipliers were adjusted accordingly to ensure no bleed-through to cross channels.

**Table 1.** List of Antibodies.

Primary antibody	Host	Dilution	Company	Secondary antibody	Reactivity
Neurofilament 68 kd (light)	Rb	1:500	Sigma	Alexa 546 gt anti-rb 1:500	Neurofilament protein light
Neurofilament 200 kd (heavy)	Rb	1:500	Sigma	Alexa 546 gt anti-rb 1:500	Neurofilament protein heavy
GFAP	Ms	1:500	Millipore	Alexa 546 gt anti-ms 1:500	Glial fibrillary acidic protein
LN	Rb	1:500	Sigma	Alexa 488 gt anti-rb 1:500	Anti-laminin
P75	Ms	1:500	Millipore	Alexa 488 gt anti-ms 1:500	P75 neurotrophin receptor
NF	Rb	1:1,000	Dako	Alexa 546 gt anti-rb 1:500	Fibronectin

GFAP: glial fibrillary acidic protein; LN: laminin; P75: nerve growth factor receptor; FN: fibronectin; ms: mouse; gt: goat; rb: rabbit.

### Statistical Analysis

Results are expressed as means ± SEM, with the statistical comparison between groups using a two-way analysis of variance (ANOVA), to determine significance. Post hoc analysis was with Sidak’s multiple comparisons and GraphPad Prism 8.0.1 software was used. Details of animal numbers are given in Materials and Methods, Results, and Figure legends.

## Results

### Cryopreserved Cells

**Figure 2.**
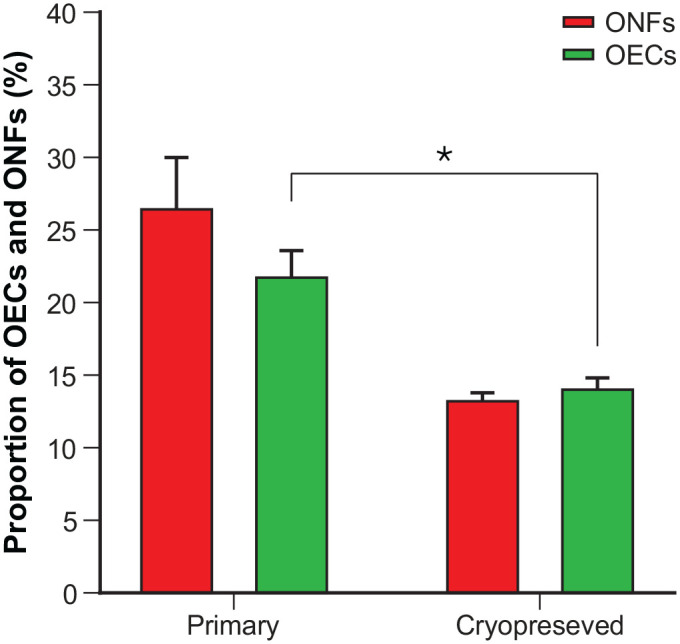
Comparison of ONF and OEC proportion in primary (*n* = 5) and cryopreserved (*n* = 5) cell cultures. Primary OB cell culture contained about 20% OEC and 25% ONF of the total population of cells, while the cryopreserved OB culture contained about 14% OEC (30% decrease) and about 13% ONF (48% decrease) which was significantly less than the primary culture. Data points were group means, and error bars: mean ± standard error of the mean. Sidak’s multiple comparison tests (* = 0.0332). ONF: olfactory nerve fibroblasts; OEC: olfactory ensheathing cells.

To investigate how cryopreservation affects the proportion of ONFs and OECs in the cell culture, the cell populations were quantified by double immunostaining for OECs (p75), ONFs (fibronectin). The OEC proportion in the primary culture was about 20%, while in the cryopreserved culture, it was around 14%; this indicates about 30% decrease in OEC population which was statistically significant (Fig. 2). However, the ONF proportion was even more significantly reduced between the primary and cryopreserved cultures, where it was at 25% in the primary culture and 13% in the cryopreserved, indicating an almost 50% reduction in ONF population.

**Figure 3.**
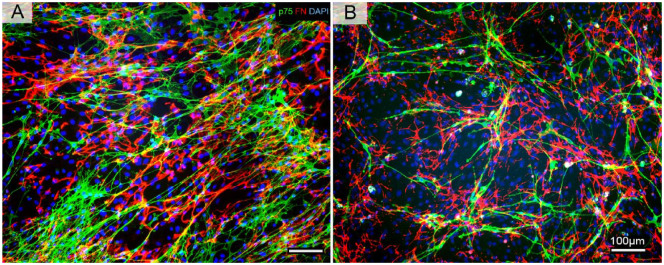
Images of double immunostaining of p75, FN, and counterstaining with DAPI. (A) Representative images of immunostaining of p75, FN, and counterstaining with DAPI to show normal primary bOEC culture after 14 days. OECs were positively stained with p75 antibody and showed spindle-shaped with small cell bodies, narrow cytoplasm with elongated, fine, and long processes. ONFs were stained with FN antibodies. Compared to OECs, their morphology appeared to be large and flat shapes with irregular shapes and short processes. (B) Representative images of immunostaining of p75, FN, and counterstaining with DAPI to show the cryopreserved bOECs after thawing from liquid nitrogen. While the OEC density appeared to be lower, the morphology was comparable to the normal primary bOEC culture. ONFs were stained with FN antibodies. Compared to OECs, their morphology appeared to be large and flat shapes with irregular shapes and short processes and was comparable to the normal bOEC culture. There were no noticeable differences in the ratio of OECs/ONFs and their morphology, but there was a significant reduction of OEC and ONF in the cryopreserved culture when compared to the primary culture. OECs stained with p75 antibody (green); ONF stained with FN antibody (red); cell nuclei stained with DAPI (blue). Scale bars: 100 µm.

To determine how the cryopreserving procedures affected the morphology of the cells, fluorescent micrographs were taken and analyzed. There are two major types of cells OECs and ONFs in the culture and they were identified by immunofluorescence staining for the P75 antibody (green) and fibronectin (FN) antibody (red). Fig. 3A showed that in the bulb culture after 14 days, the OECs appear to be spindle-shaped with small cell bodies, narrow cytoplasm, and bipolar thin and with elongated processes. Fig. 3B showed bulb culture after cryopreserving and then cultured for a further 2 days. While the OEC density appeared to be lower, the morphology was comparable to the normal culture.

### Functional Analysis

Before surgery the rats were trained to climb the cage to establish a pre-injury error score. All animals were at about 0% Grasping Error Percentage and 0 Foot Fault Score.

#### Control groups

The rats had dorsal root transection alone (*n* = 6) or the dorsal root transection with transplanted collagen gel without cells (*n* = 5).

##### Percentage error

**Figure 4.**
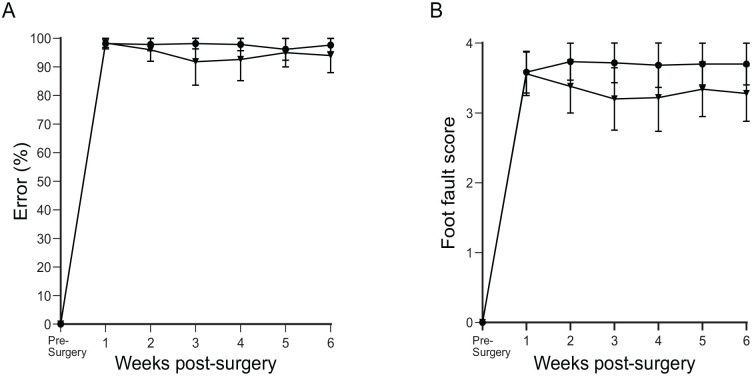
Percentage error and the foot fault score in the control groups. (A) Showing the percentage error and (B) the foot fault score over a vertical climbing task over a period of 6 weeks. Dorsal root transection alone (circle) and dorsal root transection with collagen gel transplant (inverted triangle). The data points were mean scores and error bars: mean ± standard error of the mean.

One week after surgery, the percentage error rose to 100% in both groups, meaning that every attempted grasp was a failure. Over the course of 6 weeks, this did not improve in either of the groups (Fig. 4A).

##### Foot fault

At week 1 after surgery, both groups showed a foot fault score of 3.5–4. This translated to all unsuccessful grasps, protruding past the bars as far as the elbow or as far as the axilla. Over the course of the 6 weeks, dorsal root transection alone remained at around 3.5; meanwhile, dorsal root transection with transplanted empty collagen gel appeared to slightly improve to 3; however, this still translated to unsuccessful grasps (Fig. 4B). There was no significant difference between the groups.

#### Transplant groups

The rats had the dorsal root transection and were transplanted with bOECs (*n* = 8) or CbOECs (*n* = 10) encapsulated in collagen gel.

##### Percentage error

**Figure 5.**
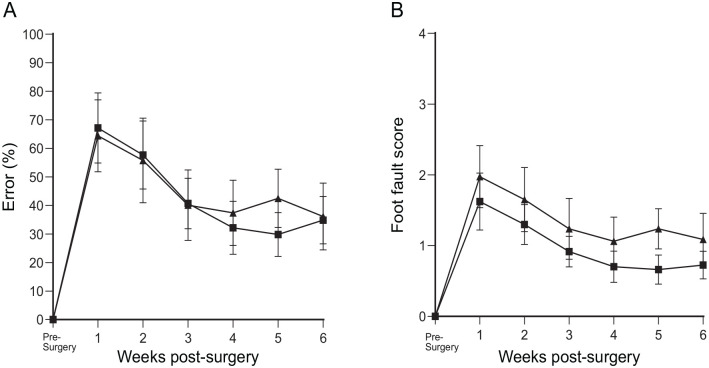
Percentage of error and the foot fault score in the transplant groups. (A) Showing the percentage error and (B) the foot fault score over a vertical climbing task over a period of 6 weeks. bOEC transplant (square) and CbOEC (triangle). The data points were mean scores, and error bars: mean ± standard error of the mean.

The results of error percentage showed that for the cryopreserved bOEC (CbOEC)-treated group, initially, the climbing error rose to 65% which already was a significant improvement over dorsal root transection alone. The error score continued to reduce and plateaued at 4 weeks to around 35%, meaning that there was an overall 65% improvement (Fig. 5A) when compared to the dorsal root transection alone (Fig. 5A). The CbOEC-treated group showed a 65% error in week 1 that gradually reduced and plateaued at around 4 weeks at around 35% just like seen in the bOEC group (Fig. 5A). There was no statistical difference between the two treated groups and there was no statistical difference between pre-surgery and 6 weeks after surgery with treatment (Fig. 5A).

##### Foot fault

The foot fault score of the treated groups was also analyzed. Both treatment groups scored between 1.5 and 2.0 (Fig. 5B). This translated to unsuccessful grasp attempts, but only protruded through the grid as far as the wrist or the paw reached grid level but did not grasp the bar. When compared to the lesion-alone control group, this was already a significant improvement. Over the next 4 weeks, the score further reduced to 1.0 where it plateaus. At this point, the grasps were either successful or the paw reached the bar not grasping it but did not protrude through it. There was no statistical difference between the two treated groups and there was no statistical difference between pre-surgery and 6 weeks after surgery with treatment (Fig. 5B).

#### Comparison between the groups

**Figure 6.**
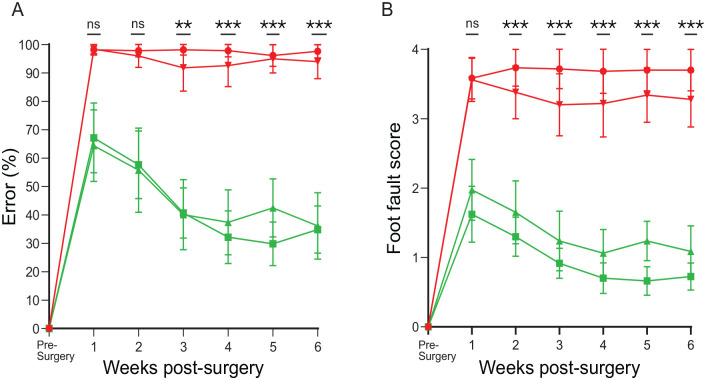
Comparison between the groups. Showing the comparison between all four groups of (A) the percentage error and (B) the foot fault score over a vertical climbing task over a period of 6 weeks. Dorsal root transection alone (circle), dorsal root transection with collagen gel transplant (inverted triangle), bOEC transplant (square), and CbOEC (triangle). The data points were mean scores, and error bars: mean ± standard error of the mean. Tukey’s multiple comparison test (**P = 0.0021; ***P = 0.0002).

The percentage error and the foot fault score on the climbing tests in the transplant groups reduced dramatically, which were statistically significant compared to the rats with dorsal root transection alone/dorsal root transection with transplanted collagen gel without the cells (Fig. 6A, B).

### Immunohistochemistry

#### Control groups

**Figure 7.**
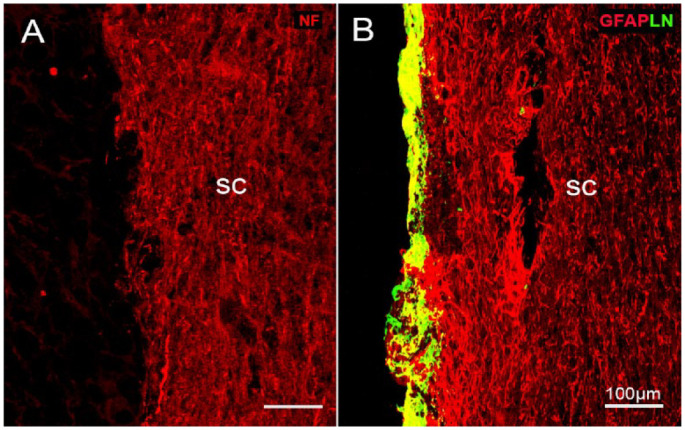
Immunostaining of NF and LN/GFAP in the control groups. (A) NF staining showing no regenerating axons presented at the DREZ. (B) Double immunostaining of LN/GFAP to show there was no outgrowth of astrocytic processes from the sc as seen in the transplant group. Cryostat horizontal plane section; sc, spinal cord; survival time: 6 weeks; scale bars: 100 µm.

There was no neurofilament (NF)-positive axons presented at the dorsal root entry zone (DREZ; Fig. 7A) in both control groups—dorsal root transection alone and dorsal root transection with transplanted collagen gel without cells.

**Figure 8.**
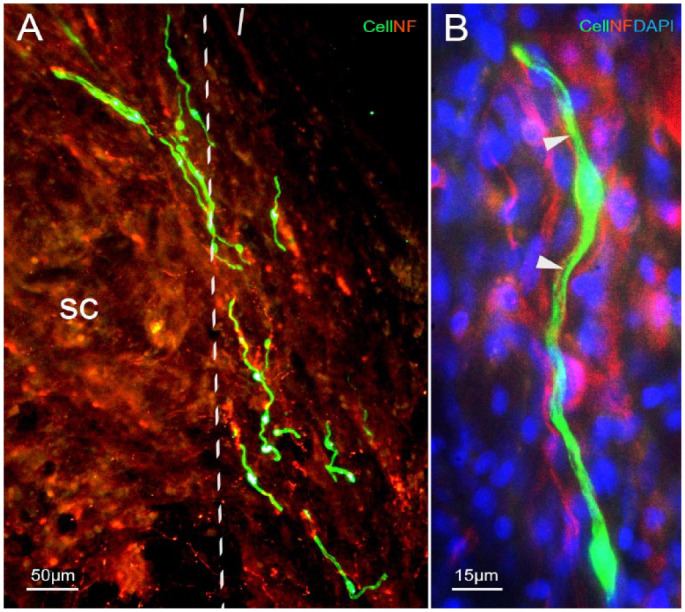
Images of immunostaining of NF and counterstaining with DAPI in the transplant group. (A) Neurofilament (NF) antibody immunostained regenerating dorsal root axons (red in A, B) at the DREZ (dotted lines). (B) High-magnification image to show the alignment and ensheathment of the transplanted OEC (green) to the regenerating axon (red, arrowheads). Blue, DAPI counterstaining. Cryostat horizontal plane section; survival time: 6 weeks; sc, spinal cord; scale bars: (A) 50 µm and (B) 15 µm.

An adjacent set of the sections were double immunostained with NF/GFAP. The staining showed that there was hardly any outgrowth of astrocytic processes from the spinal cord extending into the DREZ (Fig. 7B) as seen in the group with transplanted cells (see in Fig. 8A). An overlapped area where the peripheral nervous system (PNS) (LN antibody, green) and CNS tissue (GFAP antibody, red) interact appeared in yellow.

### Transplant Group

#### Cells/axons

**Figure 9.**
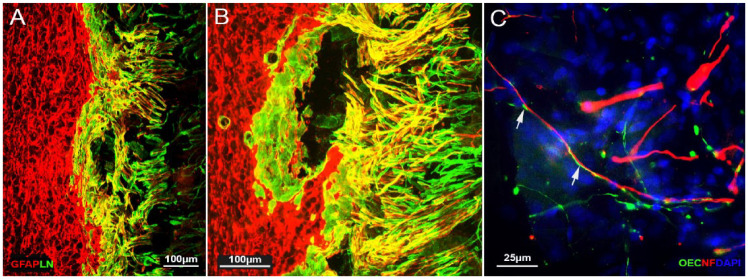
Images of immunostaining of LN/GFAP and counterstaining with DAPI in the transplant group. (A) and (B) Double immunostaining of GFAP (red) and LN (green) to show CNS/PNS interaction/ladder structure at the DREZ, and the astrocytic outgrowth from the spinal cord. (A) Low-power and (B) high-power image. (C) High-power image to show transplanted CbOECs (green) ran parallel with the astrocytic processes (white arrows) at the DREZ; counterstained with DAPI. Survival time 6 weeks. Scale bars: (A) and (B) 100 µm and (C) 25 µm.

The transplanted CbOECs were identified by green fluorescence. The cells were presented at the DREZ (Fig. 8A), and some of them migrated out of the collagen gel. The cells had elongated processes and ran parallel to the host dorsal root and dorsal column axons (Fig 8A, B). The high-magnification image (Fig. 8B) showed that transplanted CbOEC ensheathed a regeneration axon as we observed in the previous studies with transplantation of the primary cultured bulbar OECs and in the modified mucosal OECs. The transplanted cells were incorporated into the host tissue at the interface where the astrocytic processes extend out into the re-apposed dorsal roots (Fig. 9A).

#### Responses of the astrocyte at the entry zone

Double immunofluorescence staining of GFAP/LN showed that the interaction pattern at the entry zone between the peripheral and central tissues in the transplant group was markedly different from the control groups. LN+-stained PNS tissue (green) and GFAP+-stained CNS tissue (red) showed an overlapped area where the PNS and CNS tissue mingle appeared in yellow.

Multiple strands of filamentous GFAP-positive astrocytes were seen to extend their processes from the spinal cord into the transplant region (sc, Fig. 9A, B) as we observed in our previous studies with bOECs and modified mucosal OECs. The transplanted CbOECs ran parallel, ensheathed the regeneration axons, and weaved with the outgrowth of astrocytes (Fig. 9C). The merge of the astrocytic processes, the transplanted cells, and the PNS tissue formed a bridge between the severed dorsal roots and the spinal cord Fig. 9B.

## Discussion

In this study, we showed that transplantation of cryopreserved OECs cultured from the olfactory bulbs could restore loss of function in a vertical climbing test in the rat dorsal root injury model as we observed from the primary cultured bOECs in our previous studies^3,6,10,35,36^. The results showed the great potential of using auto/allograft to treat CNS injuries and are a step forward in translating the research into future clinical applications.

### Potential Use of Allograft of OECs in Clinical Applications

The autologous approach is considered the “gold standard” due to grafts’ biocompatibility with the patient. It has the advantage of avoiding the immune response that rejects the graft, as well as the use of immunosuppressants. However, this approach involves two invasive surgeries and presents the risk of poor or failure of cell production due to tissue variation from patient to patient or any unpredicted technical problems. Preparation and expansion of OECs from biopsies takes time; hence, for autologous transplantation, the surgery must wait until the cells are ready. Donor cell transplantation can be warranted for immediate use in the treatment of spinal cord injury. Although an allograft presents the risk of immune rejection, the advantage over an autograft lies in having only one surgery for the patient, ensuring the quality and quantity of the cells as well as allowing for immediate transplant of cells after injury. The time taken to transplant cells after injury could have a significant effect on functional outcomes. The longer the secondary injury is allowed to continue, the more irreparable damage is done to the spinal cord.

Our previous study showed that transplantation of xenografted mouse bulbar OECs into rats with chronic corticospinal tract lesions with daily cyclosporine dosing resulted in restored directed forepaw reaching function. Once the function had been established, cyclosporine was withdrawn, and the function was maintained although the grafted cells had been rejected. This implies that once grafted cells have acted as bridges for axon regeneration across the lesion site, their continued presence is no longer necessary for maintaining the restored function^37^. The result raises the possibility of using immune-incompatible allografted cells or cell lines, which would avoid the need for removing a patient’s olfactory bulb.

### Combining Cells With Collagen Gel

There are several biomaterials, both natural and synthetic, that have been used clinically^38–40^. We have used collagen gel to encapsulate the cells in our previous studies. Collagen is not only biocompatible, but also biodegradable and noncytotoxic^41^. It has been widely used in the clinic for a range of applications, so it is known not to introduce risk to the patient^38^. It is simple to make and encapsulate cells, requiring no specialized equipment. Once it is set, it is easy to manipulate, so it is favorable in surgery. The encapsulated cells readily proliferate within the gel and create a 3D matrix^42^. The gel itself can mold to the shape of the injury cavity to fill it; it can also be trimmed and handled to allow for more precise placement of cells. Using collagen gel, significantly fewer cells are needed which is good for clinical application. Cells transplanted with the biomaterial ensure that the cells are retained at the site of the injury and have been shown to increase cell survival; therefore, it can improve the chances of the cells migrating into and integrating with the damaged tissue^9,43^. It has been shown that OECs are highly dependent on cell–cell contacts for survival and neural regeneration^44^. It has also been shown that it affects migration, with increased migration occurring when OECs are at higher density^45^. And that the migration of OECs positively regulates axon guidance^46^. Culturing the cells in the collagen gel before transplantation, therefore, allowed the cells to form stable cell–cell connections with each other and potentially improved their regenerative abilities. Collagen gel is also readily biodegradable; it is broken down over time which reduces any potential complications such as chronic compression of newly regenerating axons^47^.

### Culturing Bio-cryopreserved Cells

An aspect of bio-cryopreserving that was evaluated as part of this study is the culturing protocols of the cryopreserved cells, especially in terms of encapsulation of the cells into collagen gel in preparation for the transplant. We found that the cells cannot be directly encapsulated after thawing as a proportion of those cells are dead. The presence of dead cells had the potential of affecting the live ones and restricting proliferation and healthy cell-to-cell communication as they took up a large volume of space and could affect the extracellular matrix. After transplantation, these dead cells would be released into the injury site which could cause an immune response that may have a negative effect on regeneration and functional recovery. Culturing cells for 2 days allows the viable cells to adhere to the dish, while the dead cells can be washed away. This ensures that only viable cells are transplanted.

### Number of Transplanted Cells in the Injury Area

In this study, we did not see as many transplanted cells (encapsulated in a collagen gel) at the entry zone as we have seen in our previous studies using the scraping method^6^. However, degree of the functional recovery observed in the present and previous studies is very similar. This may suggest that (1) the diffusion molecular and growth factors^11,48^ produced by OECs may play an important role and (2) it may not need as many transplanted cells as we used in the previous studies to restore the lost functions, as the retention and survival of these cells might be more critical.

### Primary Cell Culture Versus Cryopreserved Cells

In this study, we showed there was a lower OEC population at around 14% in the cryopreserved bulbar culture in comparison with around 20% in the primary bulb culture. However, function restoration was achieved by both transplant groups and the degree of recovery was very similar in both groups (Figs. 5 and 6). Although our study showed that cryopreservation had a negative effect on the population of OECs in culture, the results were consistent with other studies that suggest that the function of the cells is not affected^32,33^. Furthermore, the use of the biobanking means that multiple samples can be combined to ensure sufficient number of cells are embedded into the collagen gel for transplantation. This is an advantage of using the biobanking technique and is a way of overcoming a reduced OEC population in the cell culture.

This study is preliminary and only looked at cryopreserving the cells for 1 month, so further research is needed to understand how long-term cryopreservation affects the cells and whether different cryopreservation protocols would have a significant impact. Our study shows that cryopreservation for 1 month does not affect the function of the cells and leads to functional improvement in an animal study; this is encouraging and indicates the feasibility of an allograft cell therapy approach in humans.

This study showed that transplantation of cryopreserved rat olfactory bulbar cells encapsulated in collagen restored the loss of function in a dorsal root injury model as we observed previously by transplantation of primary cultured bulbar cells.

A challenging issue associated with using the patient’s own cells is that the yield of OECs can be unpredictable. In comparison, using banked cryopreserved allogeneic cells would allow for more cells obtained and pooled together which would allow for consistent transplants on demand. This method would also avoid the risks associated with autograft biopsies making the procedure safer for the patients.
